# Highly Sensitive and Stable SERS Substrate Fabricated by Co-sputtering and Atomic Layer Deposition

**DOI:** 10.1186/s11671-019-2997-8

**Published:** 2019-05-18

**Authors:** Guilin Yin, Shiheng Bai, Xinglong Tu, Zheng Li, Yanpeng Zhang, Weiming Wang, Jing Lu, Dannong He

**Affiliations:** 1 School of Material Science and Engineering, Shanghai Jiao Tong University, No. 800 Dongchuan Road, Shanghai, 200240 People’s Republic of China; 2 National Engineering Research Center for Nanotechnology, No. 28 East Jiangchuan Road, Shanghai, 200241 People’s Republic of China; 3 School of Mechanical Engineering, Shanghai Jiao Tong University, No.800 Dongchuan Road, Shanghai, 200240 People’s Republic of China

**Keywords:** Ag nanoparticles, SERS, TiO_2_, Co-sputtering, ALD

## Abstract

In this study, we develop a facile method to fabricate highly sensitive and stable surface-enhanced Raman scattering (SERS) substrate, which is realized by combining co-sputtering with atomic layer deposition technology. To accomplish the SERS substrate preparation, we firstly utilized co-sputtering silver and aluminum on glass slides to form uniform discontinuous Ag film by removing Al later, which acted as SERS active moiety and presented high sensitivity in glycerin detection. After coating an ultrathin TiO_2_ layer via atomic layer deposition (ALD), the samples could further enhance the Raman signal due to the chemical effect as well as the long-range effect of the enhanced electromagnetic field generated by the encapsulated Ag nanoparticles (NPs). Besides, the coated sample could maintain the significant enhancement in air condition for more than 30 days. The high stability is induced by TiO_2_ layer, which efficiently prevents Ag NPs from surface oxidation. This highly sensitive and stable SERS substrate might highlight the application of interface state investigation for exploring novel liquid lubricating materials.

## Introduction

Since surface-enhanced Raman scattering (SERS) was firstly reported [1], it has attracted lots of interests in detecting various analytes at extremely low concentrations due to some excellent characters such as high sensitivity, quick response, noninvasive analysis, and fingerprint recognition [2–5]. Typically, SERS contributed more and more in surface state analysis with tremendous development of in situ and real-time detection in recent years, which might open a new window for surface research [6, 7]. As a result, different materials have been explored as active SERS species, including Au, Ag, Cu, Pt, and so on [8–11]. Compared with other materials, Ag nanostructures could lead to a superior enhancement factor resulting from their unique plasmonic properties [12–14].

According to previous reports, researchers have made great efforts to enhance the SERS effect in Ag nanostructures by controlling their shapes, sizes, quantities, and arrangement on solid slides [15]. Many novel Ag nanostructures such as spheres, cubes, octahedrons, and wires have been developed to maximize their SERS capabilities and uniformities [16–19]. Furthermore, different methods have been tried to fabricate these Ag nanostructures on glass or silicon substrates, such as electron-beam lithography, reactive ion etching, immersion plating, and chemical reduction [20–22]. However, there are few reports about Ag nanostructure-based SERS substrate for interface research applied in super lubricating liquids, for challenges such as complex and costly fabrication process, easy aggregation, and rapid surface oxidation when exposed to ambient conditions. These would result in SERS activity loss of the substrate in a short time [23]. Moreover, the humidity rate of liquid super lubricate will quickly decay of the Raman signal enhancement, influencing the interface state analysis during the friction process [24, 25].

Herein, a facile method is developed to fabricate highly sensitive and stable SERS substrate based on Ag nanoparticles (NPs) by combining co-sputtering with atomic layer deposition technology for glycerin detection, which played an important role in the liquid super lubricating system [24, 25]. To obtain uniform Ag NPs on a glass slide as SERS active moiety, different content of aluminum was co-sputtered with silver firstly and was removed from glass slides by phosphate later. Noticeably, high SERS performance is realized for glycerin detection by modulating deposition power rate of silver and aluminum targets due to their significant influence on the size and distribution of Ag NPs [26, 27]. We also evaluated the stability of SERS performance by comparing the spectrum collected in different duration. In particular, after coating the active moiety Ag NPs a protectiveTiO_2_ layer via atomic layer deposition (ALD), the sample can maintain an excellent SERS performance in air condition for more than 30 days due to impeding surface oxidation and prohibit aggregation of Ag NPs. Moreover, this further enhancement effect is closely related with the TiO_2_ layer. We ascribe this to the exponential attenuation of strong electromagnetic field with the increasing thickness of the “spacer” film. The results might present a new perspective in the field of interface analysis through employing the SERS detection.

## Methods

### Fabrication of Ag NPs on Glass by Co-sputtering

Conventional glass slides (15 × 15 mm, Sail Brand) were ultrasonically cleaned orderly in acetone, ethanol, and deionized water, each for 15 min to remove the surface contaminants prior to use. The Ag NPs were deposited on pre-cleaned glass substrates at room temperature by co-sputtering silver and aluminum firstly (LLJGP-450 Magnetron Sputtering System, SKY Technology Development Co., Ltd., China). Both silver and aluminum targets are of high purity (> 99.99%) with a diameter of 60 mm (purchased from SKY Technology Development Co., Ltd., China). The base pressure of the vacuum system prior to deposition was better than 4.0 × 10^−4^ Pa, and the working argon pressure of 0.8 Pa was maintained during deposition. Noticeably, during the co-sputtering process, the power ratio was modulated by the DC magnetron sputtering power of aluminum target when the radio frequency power of the silver target was maintained at 30 W. The Al NPs were removed by immersing the glass slides in diluted phosphate acid solution (0.5 M) for 4 h. After this, the glass slides with Ag NPs were rinsed with deionized water for five times to remove absorbed phosphate or aluminum components. After drying the glass slides with nitrogen, uniform Ag NPs have been left as SERS active moiety before coating a thin protective layer. All chemicals were analytical reagents and used as received without further purification (purchased from Sinopharm Chemical Reagent Co., Ltd., China). Deionized water was obtained from the water purification system in our laboratory.

### Preparation of Protective TiO_2_ Layer via Atomic Layer Deposition

An ultrathin TiO_2_ layer was grown on the as-prepared SERS active moiety via ALD using a commercial flow-type ALD reactor (Picson-100). High-purity chemical precursors, TiCl_4_ (Alfa Aesar 99.99%), and high-purity water were employed as Ti and O sources, which were alternately pumped to the reaction chamber using ultrapure N_2_ (99.999%) as process, and carrier gas during deposition process after the chamber was maintained at a pressure of 10 hPa and 300 °C. The pulse and purge times of TiCl_4_ were 400 ms and 5 s, whereas the pulse and purge times of H_2_O were 200 ms and 8 s. The pressure in the reactor varied between 1.5 and 3 hPa during the pulsing of TiCl_4_ and H_2_O, respectively. The thickness of the TiO_2_ coating layer was controlled by deposition cycles with a growth rate of 0.04 nm per cycle.

### Characterization of Substrates and SERS Measurements

Field emission scanning electron microscope (FE-SEM, S-4800, Hitachi, Japan) was used to observe the surface morphology and structure of prepared SERS substrates. The atom information of substrates was determined by an energy dispersive spectroscopy (EDS, ORAN System SIX). UV-visible absorption spectra (Perkin Elmer: Lambda2) was performed to investigate the absorbance of the prepared Ag NPs. The SERS performance was tested on a confocal microscope Raman system (Renishaw: Invia-reflex) with a 532-nm diode laser and 1800 lines/mm grating observed through a × 50 LWD objective. Glycerol solution was used as a probing molecule during all SERS performance evaluation.

## Results and Discussion

**Fig. 1:**
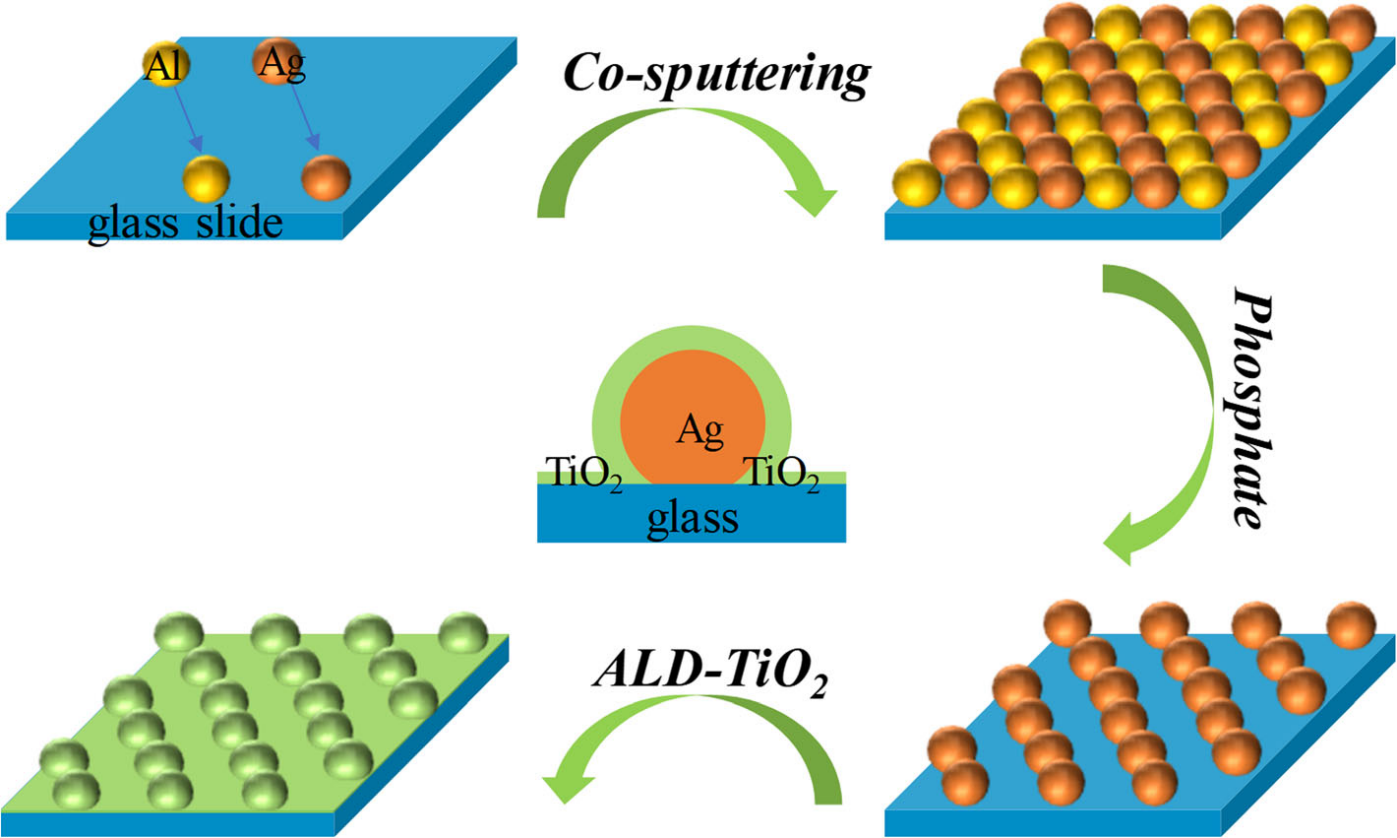
Scheme of highly sensitive SERS substrate fabricated by co-sputtering and atomic layer deposition on glass slides

In our SERS substrate, Ag NPs act as our SERS active moiety due to its high recorded enhancement factor. To prepare Ag NPs on pre-cleaned glass, aluminum was co-sputtered with silver target together firstly. Then, phosphate was used to remove Al NPs to form uniform Ag NPs on the glass. After this, an ultrathin TiO_2_ layer was coated to the Ag NP surface without pretreatment via ALD. The schematic illustration of the whole fabrication process is displayed in Fig. 1. All the preparation details have been given in the “Methods” section.

**Fig. 2:**
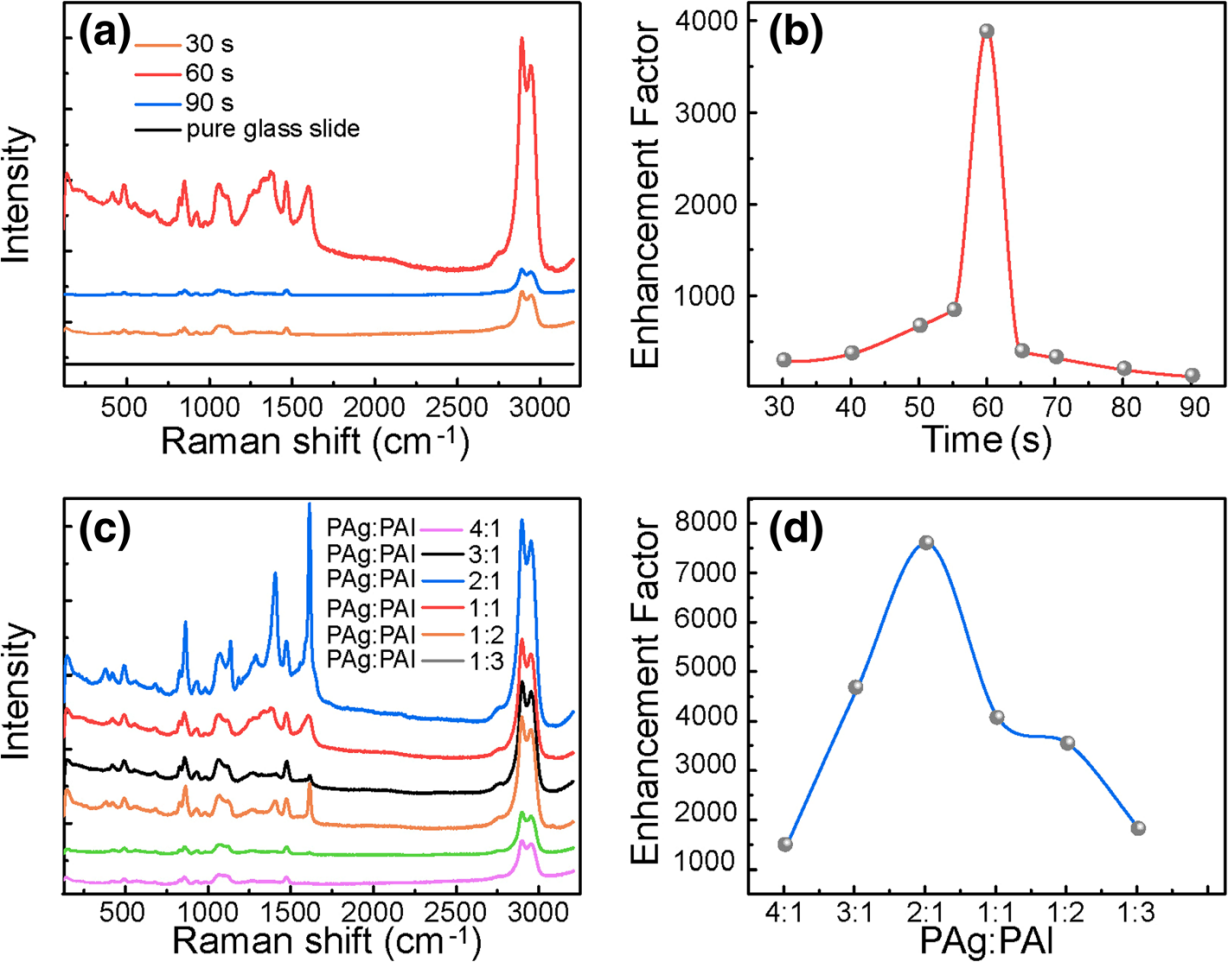
The SERS spectra of glycerin collected on substrate prepared with different (Ag, Al) co-sputtering time (**a**, **b**) and power ratio (**c**, **d**) without TiO_2_ layer

The Raman measurement was carried out by adding an equal amount of glycerol solution (usually 0.1 mL 10% glycerol solution) on all prepared SERS substrates. Noticeably, the co-sputtering time and power ratio of silver and aluminum play important roles in adjusting the size and distribution of uniform Ag NPs on the glass slide, which result in a great difference of SERS performance as well as confirming the active moiety of Ag NPs. For comparison, we investigated the influence of co-sputtering time and power ratio (silver and aluminum) on the signal enhancement factor (EF) of substrates without TiO_2_ layer, separately. As is shown in Fig. 2a and b, the glycerol Raman signals become stronger and stronger with the increasing co-sputtering time and reach a peak value at the point of 60 s with a constant 1:1 power ratio of silver and aluminum target (30 W) during the substrate deposition process. As the sputtering time continues to extend, the EF presents a sharp decrease. The deposition rate, determined by stylus profile meter on thick calibration sample, is 0.14 nm/s.

Based on this, we also display the obtained Raman spectrum and EF dependence on aluminum content in Fig. 2c and d. During the co-sputtering process, the aluminum content was modulated by adjusting the sputtering power of aluminum target with a fixed sputtering time (60 s). The Raman signal gets stronger with the increasing aluminum content first and reaches peak value at 2:1 sputtering power ratio of silver and aluminum target. The average film thickness of the sample with the best SERS performance is about 7.2 nm, calculated from the deposition rate determined the same way. Then, the EF will attenuate as the aluminum content continues to increase. The dependence of EF on both co-sputtering time and power ratio (aluminum content) is ascribed to their efficient modulation on the size and distribution of Ag NPs. As is known, both the size and distribution of Ag NPs contribute a lot to generate hot spots in local electromagnetic (EM) field among Ag NPs, which resulted in the SERS activity [15, 26–28].

**Fig. 3:**
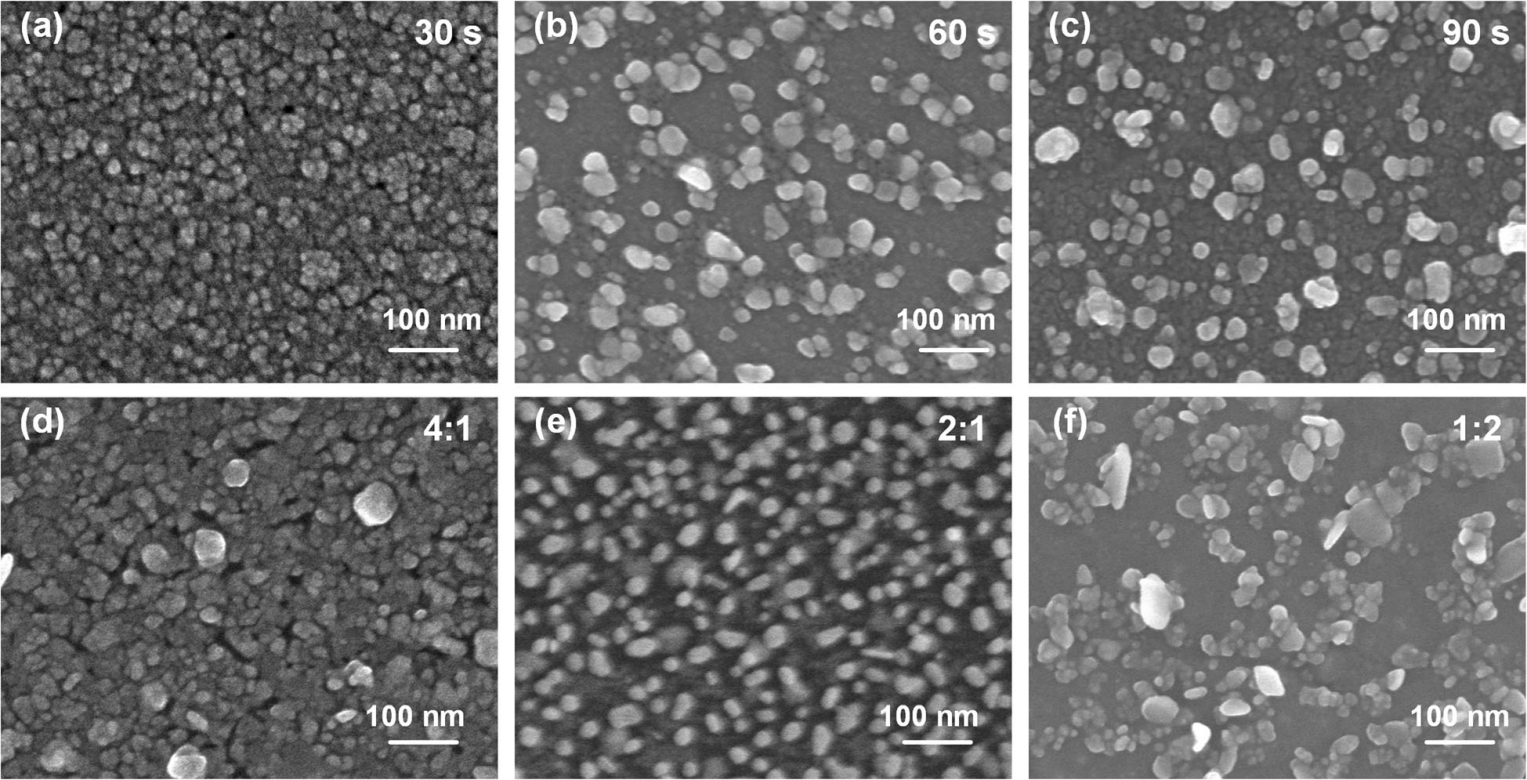
SEM images of Ag NPs prepared on glass slides at 1:1 power rate with different (Ag, Al) co-sputtering time (**a**–**c**) indicated (30 s, 60 s, 90 s, respectively) and at 60 s with different (Ag, Al) power ratio (**d**–**f**) indicated (4:1, 2:1, 1:2 respectively)

In Fig. 3, we present SEM images of Ag NPs prepared with different (Ag, Al) co-sputtering time and power ratio. The substrate with the best performance also shows a more uniform size and distribution of Ag NPs, as displayed in Fig. 3e. This also confirms their influence on SERS performance. In fact, both Ag and Al particles grow larger and faster with the augment of sputtering time and power. This is the reason why we can modulate the size and distribution of Ag NPs by co-sputtering with Al and removing it later [15, 26, 27]. It is noteworthy that compared with the samples prepared by sputtering single silver target, this co-sputtering method has significantly improved the SERS performance.

**Fig. 4:**
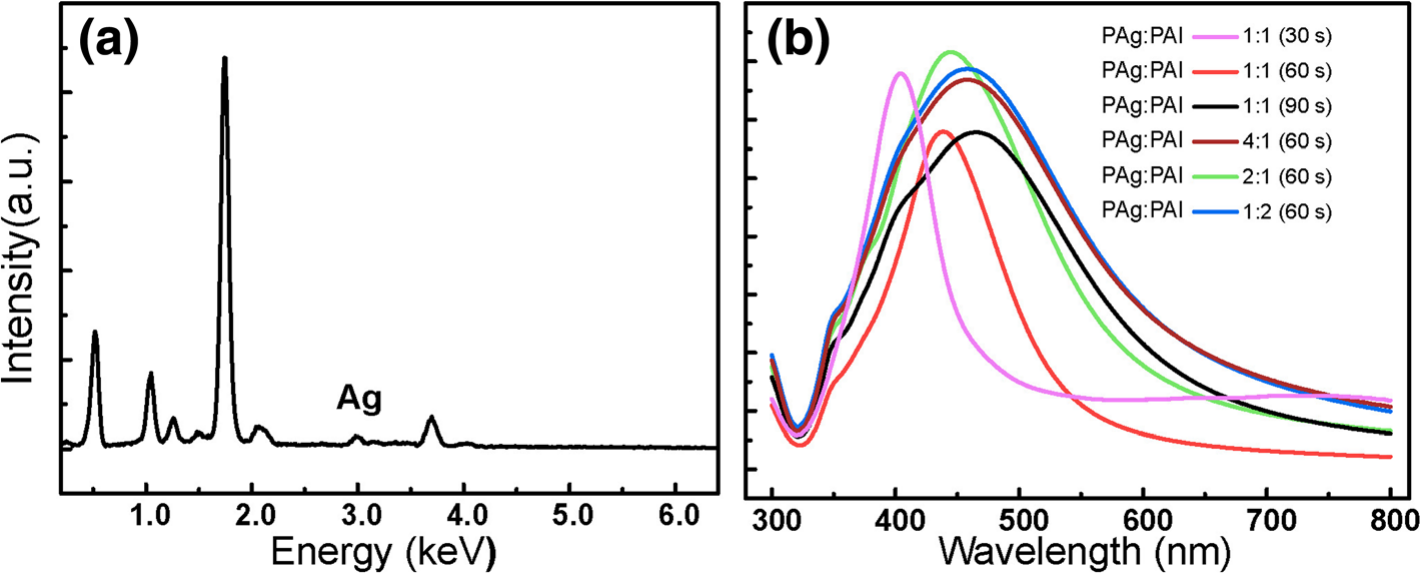
**a** EDS characterization of the sample prepared by co-sputtering (Ag, Al) targets for 60 s at 1:1 power rate. **b** UV-visible absorption spectrum of Ag NPs prepared with different co-sputtering time and power ratio

To confirm the Ag NPs as SERS active moiety, EDS characterization of the sample prepared by co-sputtering (Ag, Al) targets for 60 s at 1:1 power rate is exhibited as Fig. 4a. Besides, The UV-Vis absorption spectrum of the samples prepared by modulating co-sputtering time and (Ag, Al) power ratio is displayed in Fig. 4b. The absorption peaks varied from 404 (co-sputtering 30 s with 1:1 power ratio) to 468 nm (co-sputtering 60 s with 4:1 power ratio), which further certified the influence of size and distribution of Ag NPs on the absorption spectrum, indicating the size and distribution of Ag NP modulation during the deposition process [29].

**Fig. 5:**
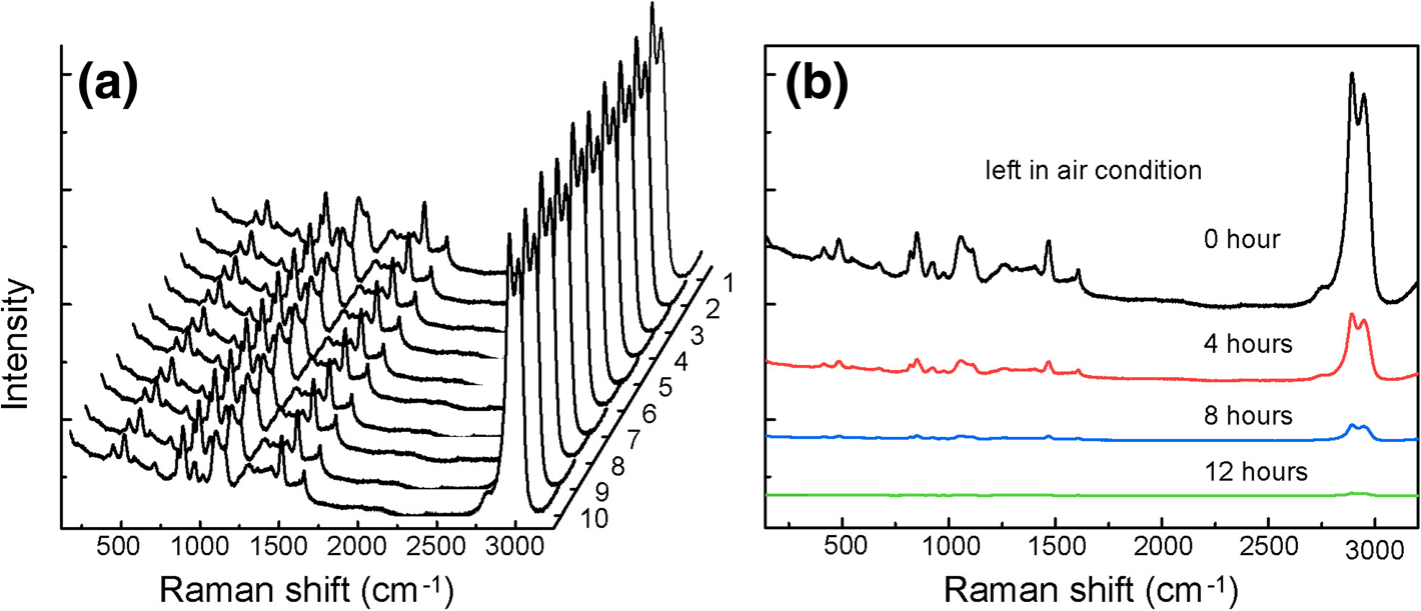
The SERS spectra of glycerin collected from **a** 10 random points on the substrate as soon as well-prepared. **b** The same position on the substrate after different time left in air condition

The uniform distribution of Ag NPs-based active moiety leads to high reproducibility of SERS performance. As is exhibited in Fig. 5a, the Raman spectra of the glycerol solution obtained from ten random spots are demonstrated. Each spot displays a distinctive Raman intensity for glycerin solution each time, confirming the excellent uniformity of the SERS performance. However, the substrate did show a serious problem in further experiments. As Fig. 5b presented, the intensity became weaker and weaker when it was left in air condition. It implied that the substrate gradually lost SERS activity, which was attributed to the easy oxidation of Ag NPs [13].

**Fig. 6:**
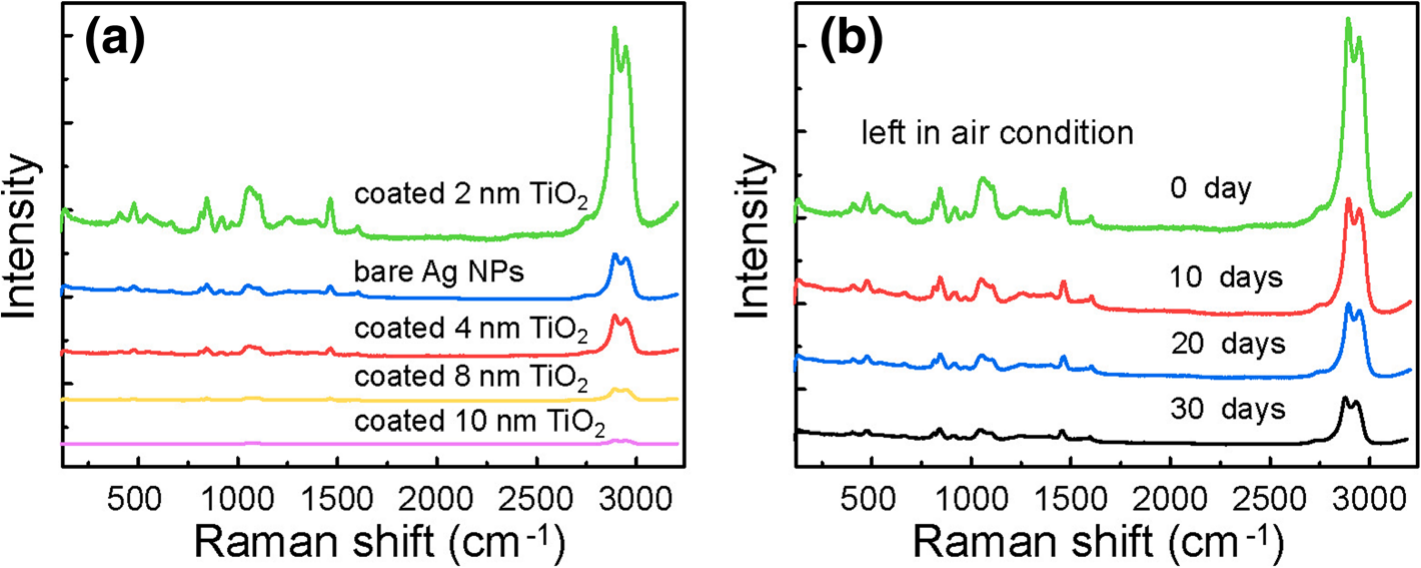
Comparison of SERS glycerin spectra collected from **a** uncoated Ag NPs on glass sides and coated with TiO_2_ of different thickness. **b** The substrate coated a 2-nm TiO_2_ film in different duration left in air condition

To improve the stability of the SERS substrate, different thin oxide layers were coated on the Ag NPs via ALD [17, 21, 30, 31]. Different from the mechanism behind Al_2_O_3_ and SiO_2_ layer, which is ascribed to the long-range effect of the enhanced electromagnetic field generated by the encapsulated Ag NPs, TiO_2_ was chosen here due to its additional contribution in SERS performance via chemical effect besides the long-range effect [32]. According to the result given in Fig. 6a, it can be clearly seen that the substrate with 2 nm TiO_2_ further enhances the Raman signal of glycerin significantly. Besides, the SERS performance is closely related with the thickness of oxide layer. As the thickness of TiO_2_ increases, the intensity of the Raman signal attenuates quickly. This is in accordance with previous reports and could be well explained by the exponential attenuation of a strong electromagnetic field with the increasing thickness of the “spacer” film [31]. The stability was evaluated by comparing the SERS performance of the substrate in different duration, which was left in air condition since it was prepared. As is shown in Fig. 6b, the SERS spectra still display distinctive intensity for glycerin solution even 30 days later, which further confirms the ultrathin TiO_2_ a protective layer.

## Conclusion

In summary, we develop a facile method to fabricate highly sensitive and stable Ag NP-based SERS substrate in glycerol detection by combining co-sputtering and ALD technology, which play an important role in super lubricating solutions. By modulating both sputtering and power ratio during the co-sputtering process, well-distributed Ag NPs on glass slides were obtained as SERS active moiety, which presented highly sensitive SERS performance. The stability of SERS substrate is significantly improved by coating an ultrathin TiO_2_ layer via ALD to impede surface oxidation and prohibit aggregation of Ag NPs. Besides, an interesting phenomenon is that the TiO_2_ layer could further enhance the Raman signal with proper thickness. We ascribe this to the contribution of chemical effect and the influence of “spacer film” on the electromagnetic field generated by the Ag NPs. This might highlight the application of SERS in interface state investigation to explore novel liquid lubricating materials.

## Abbreviations

ALD: Atomic layer deposition
FE-SEM: Field emission scanning electron microscopy
NPs: Nanoparticles
SERS: Surface-enhanced Raman scattering
